# Developing the future clinical research workforce: An immersive high school clinical research summer camp

**DOI:** 10.1017/cts.2026.10707

**Published:** 2026-02-13

**Authors:** Jessica M. Fritter, Tywan Banks, Myeshia Harmon, Smitha Sasindran, Sacha Tadros, Drew E. Spacht, Karen K. Carter, Kelly Fannin, Lauren Jones, Carolynn Thomas Jones

**Affiliations:** 1 College of Nursing, The Ohio State University, USA; 2 Clinical and Translational Science Institute, The Ohio State University, USA; 3 Columbus State Community College, USA; 4 Nationwide Children’s Hospital, USA

**Keywords:** Clinical research professionals, workforce development, high school summer camp, curriculum development, team science

## Abstract

**Introduction:**

Clinical translational research relies on clinical research professional staff, and efforts to stimulate a future workforce has included outreach to high schools.

**Methods:**

We assembled a multidisciplinary team from three institutions, leveraging expertise in education, clinical translational science, and team science to design and implement a summer camp for high school students to expose them to career opportunities in clinical research. Using backward design, we developed structured lesson plans and logistical operations. Recruitment targeted rising sophomores and juniors from Columbus, Ohio, metropolitan area high schools, with a blinded review process and no grade point averages requirement to encourage broad participation. Evaluation included pre/post assessments, facilitator feedback, and daily safety checks, with IRB exemption secured for toolkit development and dissemination.

**Results:**

Within one month, we received 100 applications from 14 schools, far exceeding expectations, and selected 34 students from 8 Columbus, Ohio, metropolitan area high schools. Of the 34 accepted, 33 participated in most elements of the program and 29 students completed all four days of camp and post-camp evaluations, with an average self-reported goal achievement score of 8.41 out of 10. Pre- and post-test results showed statistically significant increases in confidence across clinical research topics.

**Discussion/conclusion:**

The pilot summer camp for high school students, supported by in-kind contributions, successfully met its goals and led to the creation of a replicable summer camp toolkit. The camp laid a strong foundation for future offerings and collaborations, with ongoing efforts to secure funding and expand access and impact.

## Introduction

**Figure 1.**
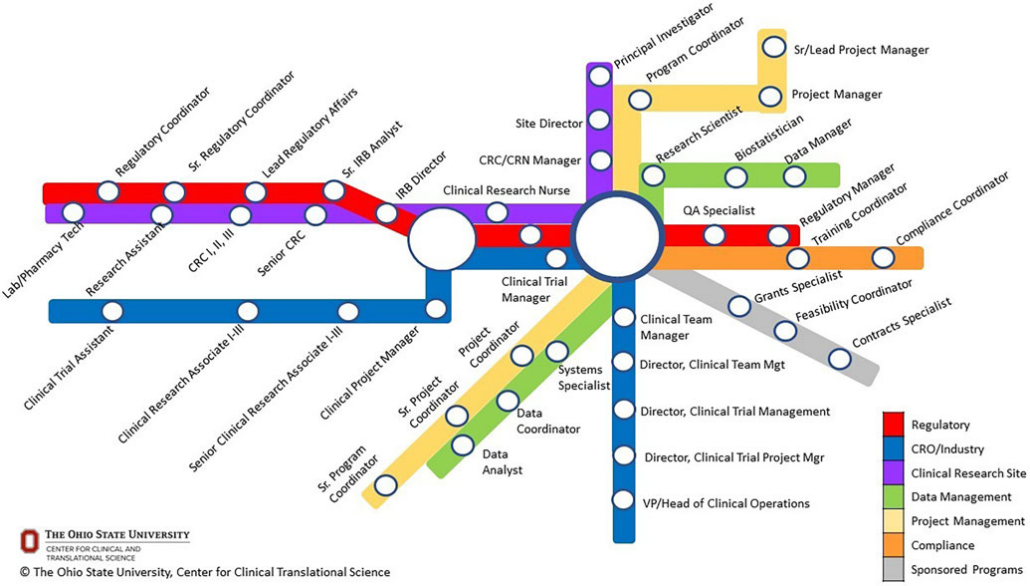
Mapping the pathway to clinical research career opportunities.

Clinical research professionals (CRPs) are key members of interdisciplinary clinical and translational science teams, encompassing a broad range of clinical, regulatory, data management, and compliance professionals. The breadth of clinical research roles is often under-appreciated by clinical research investigators and administrators, especially in the academic medical center setting, yet opportunities to enter and progress across specialty areas exist (Figure 1) [1]. A recent survey of CRPs showed that 52% of respondents (*n* = 735) felt that hiring and retention of clinical research staff is worse today than 5–10 years ago [2]. Moreover, CRPs frequently report prior lack of awareness about clinical research career options until they landed their first job, often by chance [3].

Formal educational opportunities exist for CRPs (academic certificates, associate degrees, baccalaureate, and master’s degrees) that enhance knowledge, skills, and abilities (KSAs) of novices and experts working in the field, including accreditation pathways of such programs [4]. Since the outbreak of COVID-19, several publications reflect numerous efforts to improve CRP workforce issues including exploring job titles and career ladders [5], job satisfaction issues [6], issues in onboarding and continuing education [7], defining core competencies in clinical research, and team science competencies [8]. Likewise, professional associations have also focused their attention on workforce development and studying current issues in clinical research, albeit those important efforts primarily focused on the existing CRP workforce. Due to a lack of awareness of clinical research positions, there is a notable absence of individuals seeking these roles.

To reach and build the future workforce earlier, our interdisciplinary team developed and piloted a 4-day experiential summer camp for high school students focusing on topics related to clinical research and the span of CRP roles and trajectories that exist, whether working in academic medical centers, contract research organizations, or the biotechnology industry [1]. Our team determined that expanding the knowledge base about clinical research and future careers in clinical research could have long term benefits and should commence at the high school level or sooner, not just at the baccalaureate or post-baccalaureate level. To that end, we developed a pilot curriculum and launched a summer clinical research high school camp called NEXT STEPP (STEM [Science, Technology, Engineering, and Mathematics], Technology, and Educational Pathways Program). To better facilitate future camps at our institution and others, we also drafted a toolkit curriculum based on program evaluations and team feedback that could be disseminated widely and replicated beyond the authors’ geographic area. Here, we describe the development, implementation, and evaluation of the Next STEPP Clinical Research Summer Camp pilot offering in June 2025.

## Materials and methods

### Early planning

We initially reviewed the existing summer camps at our institutions. Several high school camps focusing on Science, Technology, Engineering, and Mathematics (STEM) were offered on the campus, but most focused on laboratory, clinical, or computer-based activities exposing students to basic science, medicine, nursing, and data analytics roles. Moreover, we found many of those camps were competitive, requiring high grade point averages (GPA), which may reduce participation of students from disadvantaged backgrounds. We then searched the literature and found a paucity of publications describing high school summer camps specifically focusing on the CRP role. Through word of mouth, we identified a summer camp offered at Durham Tech Community College that informally shared their experiences with their summer STEM camp.

We formed our initial team with interested faculty and staff from The Ohio State University (OSU) Clinical Translational Science Institute (CTSI), the OSU College of Nursing (CON), Nationwide Children’s Hospital (NCH), and Columbus State Community College (CSCC) and used project management and team science approaches for planning, implementation, and evaluation of the camp. The members of this core team had experience in teaching at the high school, junior college, undergraduate, and graduate academic levels and have had extensive education in learning theory and learning content development. Moreover, team members from OSU and NCH have extensive collective knowledge in clinical translational science and interdisciplinary study management. We employed a backward design in formulating objectives, outcomes, activities, and content (in that order) [9]. We also created daily “lesson plans” for each activity so that delivery was highly organized.

Logistical operations of the camp were also planned, including institutional requirements for doing work with minors, registration, marketing, website development, and supplies for each activity. For instance, some institutions had training and background check policies and specific forms requiring parental permissions for on-site summer camp work with minor-children. To give students a wider exposure to settings, we incorporated all three institutional venues (CSCC, OSU, and NCH) with at least one day spent at each location. The camp was designed to take place Monday to Thursday from 9 am to 3 pm, with participants being dropped off and picked up at each designated location. Because we planned to create and disseminate a toolkit about the camp, including analyzing evaluations, we obtained confirmation from the OSU IRB that this quality improvement project would be exempt from review. Orientation packet materials for camp participants and parents were developed, including consent materials from each institution that required parental signatures, and a welcome video for dissemination once camp participants were selected. Live zoom orientations were also scheduled with parents and camp participants.

### Camp participant recruitment

There are 80 public high schools in the Columbus Metropolitan area. Of those 21 (26.25%) are city schools. The camp population was recruited from Columbus, Ohio, metropolitan area high schools through outreach and information provided to district leaders as well as school principals and counselors, including a hyperlink to the camp website. To be eligible for participation, inclusion required camp participants be rising sophomores or juniors. Applicants were required to write a brief essay explaining why they were interested in the camp and to affirm that if they were selected, they would attend all four days. GPA was not included in the inclusion criteria because we wanted to enhance participation in the camp and not limit potential camp participants as this could be motivational for future academic achievement. The camp was offered free of charge. Individuals from the central team reviewed applicants independently and scored de-identified materials (blinded on name, gender, race, and ethnicity). Scoring criteria were based on grade level, and personal statements on “Why is this summer camp of interest to you?” As a pilot, we planned to accept 30 students to demonstrate feasibility, future sustainability, and resource planning.

### Camp curriculum

#### Learning theory

**Figure 2.**
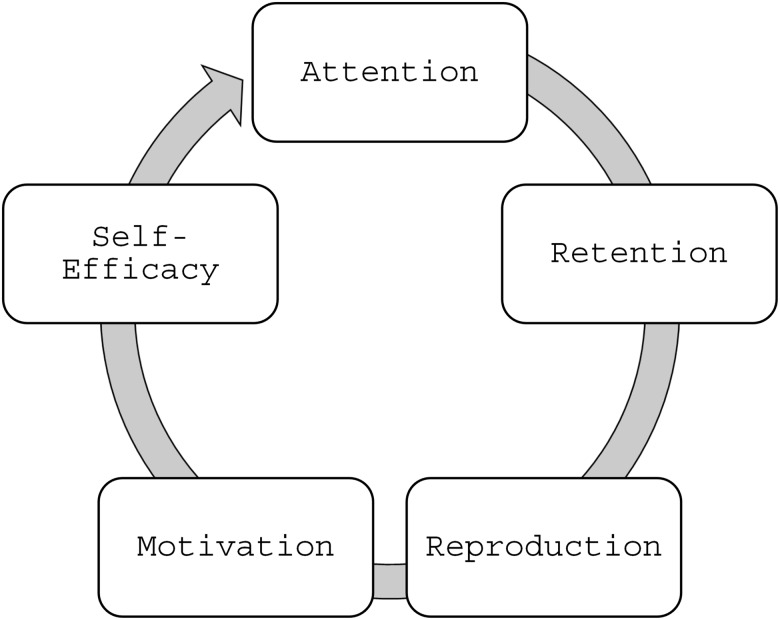
Bandura social learning and self-efficacy cycle.

To ensure the curriculum was established for the adolescent learner, and because we wanted the camp participants to co-create in groups, we applied Bandura’s 4 Elements of Social Learning Theory in planning the curriculum: (1) *Attention* (a lesson must engage students sufficiently to hold their attention; (2) *Retention* (learning activities promote retention of content, by engaging delivery, context, and opportunities to interact with the subject matter) (3) *Reproduction* (students are able to practice what they are learning through meaningful activities); and (4) *Motivation* (students will see benefits of the new things they learned, are motivated to continue learning, and assimilate toward visioning a future in clinical research) [10]. We also applied Bandura’s Self-efficacy Theory when developing activities, ensuring they emphasized teams succeeding together, with positive encouragement from facilitators, can further learning motivation and self-efficacy [11]. This pedagogy leads to a cycle of influence that could motivate students to lean into STEM and clinical research content in the future and begin to visualize themselves in future clinical research roles (Figure 2). We also employed a variety of teaching approaches to facilitate learning in these camp participants.

#### Camp aims

Overall, the aims of this pilot for the camp were to:

1. Create a summer camp to expand awareness about clinical research and the profession to high school students (camp participants).
2. Facilitate learning experience for camp participants to learn and demonstrate the basics of clinical research and clinical research operations.
3. Expose camp participants to a variety of CRPs working in different clinical research settings, including discussion with a study participant and their caregiver.
4. Develop a toolkit that can be replicated locally and by other institutions/organizations to build the future clinical research workforce.

The camp navigated groups of camp participants through experiences whereby they would formulate a research question, develop a study protocol, and present and defend their study plan through a poster presentation. They were also exposed to interactive activities related to subject recruitment, informed consent, gaining insights on participant experiences, and network with professionals working in clinical research to better vision future career paths. We developed daily learning objectives mapped to clinical trial competency domains, type of learning (KSA domains) and delivery method to make certain that topics align at a fundamental learning level learning [12,13]. We also exposed camp participants to Translational Science Principles: focusing on unmet needs, seeking generalizable solutions, engagement in team science and boundary-crossing partnerships, bold and rigorous approaches, efficiency and speed, and using creativity and innovation [14]. To maintain motivation, camp participant experiences with didactic content facilitators included interactive discussion prompts to maintain camp participant interest and accessible contextualization. Table 1 describes the daily learning objectives, alignment with the JTF competencies, type of KSAs (knowledge, skill, and ability), and content delivery method (didactic discussion [DD], group interactive participation [GP], or individualized reflection).

**Table 1.** Daily camp learning objectives

	JTF domains	KSA domains	Delivery
**Day 1: Location: CSCC**			
**Apply** team science to form and participate in a small group and larger camp community.	8	S, A	GP
** Calculate** and present basic descriptive statistics for their small group from the icebreaker survey.	6	K, S	GP
** Describe** a basic understanding of clinical research purpose and methods to answer a research question.	1	K	DD
** Explore** the process of new product research and approval for marketing.	3	K	DD
** Explain** the importance of bioethics and quality processes in conducting clinical research.	2, 4, 5	K, A	DD
** Develop** a PICOT question for a future study.	1	S	GP
** Execute** hands on laboratory techniques: micro-pipetting and microbiology culture plating.	1	S	GP
** Maintain** reflection log of today’s experiences.	7	A	IR
**Day 2: Location: OSU**			
** Discuss** the importance and impact of clinical research in solving different health problems.	1	K, A	DD
** Develop** a clinical research protocol from a PICOT question.	1	K, S	GP
** Explain** study participant recruitment for a group protocol.	2, 4	A	DD, GP
** Discuss** elements of informed consent.	4	K, A	DD
** Demonstrate** delivering informed consent in mock practice sessions.	2, 4	S	GP
** Witness** the application of technology for innovative health research and practice.	1, 3	K	GP
** Maintain** reflection log of today’s experiences.	7	A	IR
**Day 3: Location: NCH**			
** Explore** pediatric research topics and impacts.	1, 2, 3	K	DD
** Observe** the use of technology in clinical research settings: 3-D printing and patient simulation laboratories.	1, 3	K	GP
** Gain** a perspective of the participant and caregiver experience in a genetic pediatric clinical trial.	2	A	DD/GP
** Create** a scientific poster to describe group study question and protocol.	1, 7, 8	S	GP
** Maintain** reflection log of today’s experiences.	7	A	IR
**Day 4: Location: CSCC**			
** Dialogue** with a panel of clinical research professionals working across the clinical research enterprise about their career progression in clinical research.	7, 8	K, S	DD
** Orally** present group protocols in a poster presentation.	8	S	GP
** Critique** results from microbiology experiments on Day 1.	1	K	DD/GP
** Reflect** on daily activities and future career opportunities in clinical research and experiences in the camp.	7	A	IR

K = knowledge; S = skill; A = ability/attitude; DD = didactic discussion; GP = group participation; IR = individualized reflection; PICOT = Population, Intervention, Comparison/Control, Outcome, Time.

#### Lesson plans

To ensure a structured and enriching experience for the participants, we crafted detailed and timed lesson plans for each daily activity of the camp. These plans included: activity titles, primary and assisting facilitators involved, objectives, a motivational introduction to contextualize content, the content outline, special preparations (if required), supply list (if required), activity instructions (if required), and a debrief moment at conclusion. As plans were developed, we used a backwards design approach that initially focused on learning objectives and outcomes, building the content from there [9].

A binder was created for camp participants that included: daily activity outlines (including lesson plans), slide handouts for notetaking, reflective journal for the day, a checklist of lessons learned (by learning objective), and activity handouts and worksheets. The handouts and worksheets included materials on: (a) collecting and analyzing survey results, (b) writing a PICOT (Patient/Population/Problem, Intervention, Comparison, Outcome, and Time) question, (c) structuring a brief protocol synopsis, and (d) template for a scientific poster.

This thorough planning provided that the camp activities were well organized and impactful. Each day, lead facilitators opened the camp with motivational discussions on the day’s topics and activity plan, including reflections on the prior day’s activities. All didactic slide presentations were deliberately concise and included publicly available short video clips and opportunities to pause and discuss prompts.

### Camp participant learning teams

Camp participants were divided in advance into smaller working groups that would sit together, helping to establish a camp micro-community and teams that worked together on group projects. Therefore, groups (teams) were assigned to seating areas in advance to facilitate group work. This structured approach also served to conserve time and ensure camp participants did not drift away. Groups also traveled together to special activities: laboratories, innovation studios, skills labs, and facility tours on a schedule. Snacks and lunch were provided each day, with labeling to accommodate dietary restrictions. If there was not a working lunch activity, students were free to drift to other teams to consume lunch.

### Evaluation

We designed evaluations including a pre-test, post-test assessment of self-perceived clinical research knowledge and skills. The pre-test was delivered via a Qualtrics survey link on Day 1 before the camp commenced. The survey included eight questions where the camp participants rated their confidence in performing clinical research activities on a scale of 0 (not at all confident) to 10 (extremely confident). Camp participants used their smart phones to access the QR code or URL provided. At the end of Day 4, camp participants were asked to again go to the post-test survey links provided to rate the same activities on a scale of 0 to 10. We used a two-sample *t*-test (Welch’s *t*-test) to compare pre- and post- test results. Moreover, we included mixed methods evaluation of camp participation satisfaction of lessons learned, objectives, content, activity experiences, and setting, including open-ended qualitative feedback. A five-point scale was used (1 = extremely dissatisfied; 2 = slightly dissatisfied, 3 = neither satisfied nor dissatisfied; 4 = moderately satisfied; 5 = extremely satisfied) and students were given opportunities to answer open-ended questions in free text. Furthermore, camp facilitators were also included in evaluating the program through an anonymous survey to collect their perceptions of what went well and suggested improvements. Finally, we conducted daily safety evaluations. Being aware of the risks associated with bringing minor adolescents to our campuses, we built a daily safety evaluation to ensure we maintained safety protocols and closed any detected gaps.

## Results

Over a one-month recruitment period of the 80 schools contacted, we had 100 applicants from 14 high schools (17.5% of schools) including one unidentified school, much higher than anticipated; however, we had no applicants from the city schools, despite recruitment efforts. In our application, we did not collect family income, personal zip codes, race, ethnicity, or grade point average (GPA). Therefore, we are unable to report accurate data on those demographics. Camp participants did provide the high school’s name and the rising grade level (Sophomore, Junior). Reviewers accepted 34 students from 8 high schools in the metropolitan Columbus area using a scoring system.

Of the 34 accepted camp participants, 32 camp participants attended Day 1 (see Table 2). One camp participant emailed withdrawing their acceptance before the camp started, we had one no show on Day 1. During the camp, three camp participants dropped out due to illnesses. A total of 29 camp participants from the 6 groups completed the full four days of the camp and associated evaluation activities.

**Table 2.** Camp participant demographics

	Gender (*n*)		Grade (*n*)		Total	
	M	F	RS	RJ	*n*	%
School A	0	1	1	0	1	3
School B	0	1	0	1	1	3
School C	0	1	0	1	1	3
School D	2	11	6	7	13	45
School E	1	0	0	1	1	3
School F	0	1	1	0	1	3
School G	1	5	5	1	6	21
School H	2	3	3	2	5	17
Total *n* (%)	21%	79%	55%	45%	29	100

M = males; F = females; RS = rising sophomores; RJ = rising juniors.

### Camp participant goals

Camp participants developed personal goals for the camp before commencing Day 1. On Day 4 they evaluated achieving their initial goal. Twenty-nine camp participants completed this evaluation where the average score for “how well did I achieve the goal I set for attending the camp on a scale of 0 to 10” was 8.41 (range 4, 10).

### Pre-test/post-tests

Results of the pre-test and post-tests are summarized in Table 3. Overall, there were significant improvements in camp participants’ confidence in clinical research topics across the four days.

**Table 3.** Mean pre- and post-test results (Scale 0–10)

Clinical research skills	Pre	Post	*p*-Value
Developing a research question.	5.41	8.66	< 0.0005
Performing a descriptive analysis of survey data.	4.65	7.52	< 0.0001
Performing laboratory activities such as micro-pipetting and plating microorganisms on agar.	4.03	9.21	< 0.0001
Creating a simple study protocol.	4.94	8.52	< 0.0001
Identifying the needs of vulnerable research participants.	5	8.59	< 0.0001
Understanding different clinical research career professions.	4.94	8.55	< 0.0001
Study participant recruitment and informed consent.	4.79	8.79	< 0.0001
Summarizing a research project for a poster presentation.	6.56	9	< 0.0001

### Daily satisfaction scores from camp participation

Only 22 of the camp participants completed the satisfaction survey for each day, including satisfaction with content, materials, and facilitators are shown in Table 4.

**Table 4.** Daily mean camp participant satisfaction scores

Daily activities	Mean satisfaction score (*n* = 22)
(1, very unsatisfied; 5, very satisfied)	
**Day 1: Location: CSCC**	
Analysis of a survey and creating a research question	4.41
Content provided by facilitators	4.50
Lab activities: pipetting and culture plating	4.95
Materials provided	4.91
Activity facilitators	4.68
**Day 2: Location: OSU**	
Activity: developing a study protocol	4.32
Content provided by facilitators	4.32
Tour to VR and informed consent lab activities	3.95
Materials provided	4.41
Facilitators	4.55
**Day 3: Location: NCH**	
Activity: learning about pediatric research	4.86
Content provided by facilitators	4.82
Tour to 3-D printing and clinical simulation labs	4.73
Materials provided	4.59
Facilitators	4.82
**Day 4: Location: CSCC**	
Activity: career panels	4.55
Content provided by facilitators	4.59
Activity: poster development and presentation	3.82
Materials provided	4.59
Facilitators	4.64

During the application process, we collected information about camp participant food sensitivities, allergies, and preferences; however, some of the caterers did not adequately label foods that were specific (e.g., gluten free) and camp participants were reluctant to eat those. Camp participants were very aware of their own dietary needs and expressed dissatisfaction when some of the food selections were ambiguous.

## Discussion

We developed, facilitated, and evaluated a pilot 4-day high school camp that provided content related to clinical research. Our main purpose in developing the camp was to expand awareness of clinical research career roles at the secondary school level to enhance the future workforce in clinical research. This initial year of our summer camp was met with enthusiasm from high schools as evidenced by the 100 applicants received in a 30-day period. Forty-five percent of the camp population came from one high school. This high school is in the fourth largest school district in the state of Ohio. We sent camp information to multiple school districts and we did not track how often each school district marketed the camp to their student body [15]. We believe more in person recruitment and presentations about the camp, plus inclusion of stipends could be used in the future enabling more students to apply. We hope to identify barriers to students applying to the summer camp in our next cycle of applications. Future grants are being sought to increase camp sessions and offer such outreach and incentives. Although we were not equipped to accept all applicants, this pilot camp endeavor enabled us to create a summer camp toolkit that could be disseminated for use at other locations and shorten planning time and potentially offer more than one session in a future offering. We plan to update the toolkit as we implement new improvements like updated sessions and activities into the summer camp. By including professionals across the local clinical research enterprise as guest panelists we attracted future collaborators who expressed eagerness to sponsor and participate in future camps. These factors will aid in future sustainability and growth of this community outreach program.

The camp participants particularly enjoyed learning about pediatric research on Day 3 through a discussion led by a clinical research nurse, a patient who had experienced successful gene therapy for muscular dystrophy and their mother (caregiver). The patient and their mother remained during lunch, and the camp participants enjoyed ongoing interaction, especially with the pediatric patient who has survived to be in their early 20s because of the life saving gene therapy. This humanized the clinical research experience and was a significant motivator for future engagement in clinical research.

### Limitations

This camp was an initial pilot offering that was funded through in-kind donations from The Ohio State University’s Clinical Translational Science Institute and College of Nursing, Nationwide Children’s Hospital, and the Columbus State Foundation of the Columbus State Community College, and The Amgen Biotech Experience. The team of primary facilitators from each institution dedicated substantial time to developing the content for the camp, in weekly Zoom meetings, and during the 4-day camp. Moreover, execution of the camp would not have happened without additional volunteers. Because this work was not part of standard job assignments, this was a passion project that would require future funding to remain sustainable and increase future camp offerings.

We experienced a few challenges during the camp. These included unexpected staff absences, survey mishaps, and unforeseen parent involvement. On Day 4, camp participants and facilitators did not anticipate the full participation of the parents and career panel during the poster presentations. The camp participants held their ground when multiple parents and professional attendees asked challenging questions about their proposed studies. Facilitators recognized that a failure to prepare the camp participants for being on the spot and fielding comments could have dampened their experience and had a negative impact. Nevertheless, camp participants were resilient and enthusiastic during their presentations. They were proud of their accomplishments and stayed past dismissal while continuing to network with the career panel and facilitators.

We also worried that having camp participants travel to three locations (OSU, NCH, and CSCC) might cause confusion and safety issues for camp participants and parents; however, camp participants’ satisfaction scores for the three locations were consistently high (average 4.64 out of 5), and parents did not mind dropping them off and picking them up at different locations each day.

Facilitator feedback during camp debriefing was very positive. The amount of work that went into planning the camp, especially the development of the toolkit, will facilitate replicating the camp in future sessions. We did have a safety concern, in that camp participant departures from one location was confusing, and one camp participant was picked up late after most facilitators had left. Additionally, it was hard to tell our camp participants from the college students on the campus; therefore, camp t-shirts could help us better identify stragglers and ensure safety. Having dedicated team members assigned to entry and exit procedures is also an additional safety feature we would recommend to other groups.

The camp may benefit from including graduate students and principal investigators to address the camp participants. Stanford University features a highly effective high school outreach program entitled “Future Advancers of Science and Technology (FAST)” that is offered to three local high schools and meets two Saturdays a month (12 to 5 pm) during the school year. Their camp curriculum is strictly facilitated by graduate students who sign up to facilitate specific sessions [16]. Furthermore, a team science ambassador program has been described that occurs over a two-year period during the academic year and incorporates internship opportunities. In addition to having facilitator mentors in science, this program includes “near peers” who are students who attended the program in the prior year [17]. Though our program is a short-term (4-day), nonresidential summer camp, it may be worthy of considering embedding “near peers” from this year’s offering into workgroups in future summer camps. The camp could also offer graduate students’ opportunities to serve in a community outreach activity. Moreover, using graduate students to assist with facilitation could improve the camp participant experience and put less burden on staff.

The Next STEPP team leaders are in the process of seeking additional external funding to ensure a lower financial burden across our collective departments and/or institutions, and to expand the camp to two summer offering dates in future years.

### Unexpected outcome

An unexpected positive outcome of the camp was that two camp participants developed a new high school club entitled “CURE (Clinical Understanding, Research, and Exploration),” which is designed to broaden awareness of clinical research, bring in speakers and read articles. The CURE Club ultimately plans to generate a competition where students will research a disease and drug, review clinical trial results, and identify the controversies associated with the trial and/or drug study intervention. Outcomes like this are part of the value of Next STEPP and increased the culture of innovation that our summer camp fostered.

## Conclusion

The Next STEPP summer clinical research camp was a successful opportunity to expose high school students to clinical research and the varied careers that exist in the clinical research enterprise. The curriculum for the camp was well organized and improved using evaluation feedback from camp participants and facilitators, culminating in a PDF Toolkit that includes daily lesson plans and assessment tools. As part of our efforts to improve the future workforce, we are using this process to lay the groundwork for future Next STEPP summer camp offerings locally. By providing the Toolkit on our OSU Clinical Translational Science Institute and College of Nursing websites [18,19], we hope to reduce planning and production time for others who may wish to replicate it within their own institutions to impact the future clinical research workforce.

## References

[1] Fritter J , Jones CT. Mapping the pathway to take control of your clinical research career. Clin Res. 2023;37(5):7–15. PMC11172395 38873692

[2] ACRP. Voices from the front lines: Insights from the workforce on transforming the clinical research enterprise- past, present and future. Arlington, VA: ACRP. 2025 (https://acrpnet.org/insights/white-papers/voices-from-the-frontlines) Accessed September 10, 2015.

[3] Freel SA , Snyder DC , Bastarache K , et al. Now is the time to fix the clinical research workforce crisis. Clin Trials. 2023;20:457–462. doi: 10.1177/17407745231177885. 37264897 PMC10504806

[4] Sonstein SA , Silva H , Jones CT , Bierer BE. Education and training of clinical research professionals and the evolution of the Joint Task Force for Clinical Trial Competency. Front Pharmacol. 2024;15:1291675. doi: 10.3389/fphar.2024.1291675. 38303986 PMC10830830

[5] Stroo M , Asfaw K , Deeter C , et al. Impact of implementing a competency-based job framework for clinical research professionals on employee turnover. J Clin Transl Sci. 2020;4:331–335. doi: 10.1017/cts.2020.22. 33244414 PMC7681128

[6] Knapke JM , Harris SK , Kues J , et al. Results of a pilot survey assessing job satisfaction factors for clinical research professionals employed at academic medical centers (Part II). J Clin Transl Sci. 2025;9:e55. doi: 10.1017/cts.2025.34. 40201634 PMC11975783

[7] Knapke JM , Jenkerson M , Tsao P , et al. Academic medical center clinical research professional workforce: Part 2 - issues in staff onboarding and professional development. J Clin Transl Sci. 2022;6:e81. doi: 10.1017/cts.2022.412. 35949655 PMC9305080

[8] Mendell A , Fritter J , Helm S , et al. Team science competencies for clinical research professionals: a multi-leveled Delphi approach. J Clin Transl Sci. 2024;8:e112. doi: 10.1017/cts.2024.509. 39655002 PMC11626577

[9] Rinaldi K , Messer M , Hanson A , Chan J. Utilization of backward design in health professional education: a rapid review. J Prof Nurs. 2025;58:31–38. doi: 10.1016/j.profnurs.2025.02.004. 40368493

[10] Bandura A. Social Learning Theory. Englewood Cliffs, NJ: Prentice Hall; 1977.

[11] Bandura A. Self-efficacy: Toward a unifying theory of behavior change. Psycology Reviews. 1977;84:1910215. 10.1037//0033-295x.84.2.191 847061

[12] Sonstein SA , Seltzer J , Li R , Jones CT , Silva H , Daemen E. Moving from compliance to competency: A harmonized core competency framework for the clinical research professional. Clin Res. 2014;28:17–23.

[13] Sonstein S , Brouwer RN , Gluck W , et al. Leveling the joint task force core competencies for clinical research professionals. Ther Innov Regul Sci. 2020;54:1–20. doi: 10.1007/s43441-019-00024-2. 32008235

[14] Faupel-Badger JM , Vogel AL , Austin CP , Rutter JL. Advancing translational science education. Clin Transl Sci. 2022;15:2555–2566. doi: 10.1111/cts.13390. 36045637 PMC9652430

[15] Behrens C. Olentangy schools is largest district in Ohio to earn State’s top report card rating. The Columbus Dispatch. 2025 (https://www.dispatch.com/story/news/education/2025/09/17/olentangy-is-largest-ohio-district-to-earn-top-report-card-rating/86182885007/) Accessed October 13, 2025.

[16] Stanford University. FAST- future advancers of science and technology. 2025 (https://fast.stanford.edu) Accessed October 13, 2025.

[17] Browning BD , Glover JS , Meredith LR , et al. A three-tiered mentorship approach for supporting high school students interested in science, technology, engineering, and mathematics (STEM) careers. J Clin Transl Sci. 2025;9:e54. doi: 10.1017/cts.2025.19. 40276494 PMC12018214

[18] OSU Clinical Translational Science Institute. Next STEPP clinical research summer camp. 2025 (https://ctsi.osu.edu/career-development/clinical-research-professionals/next-stepp-clinical-research-summer-camp) Accessed October 13, 2025.

[19] OSU School of Nursing. Next STEPP clinical research summer camp. 2025 (https://nursing.osu.edu/research/next-stepp-clinical-research-summer-camp) Accessed October 13, 2025.

